# Evolution of traditional Chinese medicine registration review and approval policies: research based on the LDA topic model

**DOI:** 10.3389/fpubh.2025.1655636

**Published:** 2025-10-01

**Authors:** Kaidi Lu, Ming Xie, Wanping Sun, Yiming Liu

**Affiliations:** School of Pharmacy, Liaoning University of Traditional Chinese Medicine, Dalian, China

**Keywords:** Chinese traditional medicine, herbal medicine, phytotherapy, policy making, public policy

## Abstract

**Introduction:**

The economic expansion of the Traditional Chinese Medicine (TCM) industry has prompted the Chinese government to introduce a series of policies focused on the registration review and approval of TCM. These policies aim to provide scientific and practical guidance for the innovation, protection, and progress of TCM. Although scholars have conducted detailed studies on the quantitative assessment of Traditional Chinese Medicine Registration Review and Approval Policies(TCMRAPs), Research on the evolution analysis of these policies is still relatively lacking.

**Methods:**

The evolution of TCMRAPs was analyzed using the Latent Dirichlet Allocation (LDA) topic model.

**Results:**

The results show that the scope of concern of the TCMRAPs in China is vast. TCMRAPs demonstrate their uniqueness at each stage, and over time, they have exhibited a development trend from a basic framework to standardization and refinement.

**Discussion:**

This study provides a reference for the subsequent formulation of TCMRAPs in China. Also, it offers an assessment method and theoretical reference for other countries in formulating their own policies for the drugs.

## 1 Introduction

The wisdom of TCM has been built over thousands of years (1). As a valuable part of Chinese cultural heritage, it encompasses rich theoretical knowledge and extensive clinical experience (2, 3). In recent years, China has developed a comprehensive regulatory framework for new TCM, managed by the National Medical Products Administration (NMPA) (4). This framework regulates individuals and companies through specific TCM registration policies and procedures (5). The legal foundation for this regulatory system is the Drug Administration Law of the People’s Republic of China, which outlines detailed regulations for the approval, production, marketing, and use of new TCM drugs (6). The developmental trajectory of TCMRAPs vividly reflects the government’s profound emphasis and robust support for the TCM industry, as well as policymakers’ deep insight into and precise grasp of TCM development laws (7). A thorough review of this policy evolution helps us better understand the logic and direction of policy formulation, providing valuable experience for subsequent policy development.

The LDA topic model is a generative probabilistic model designed to uncover topics within a corpus based on the probability of various words occurring together (8). In this model, each record represents a distribution of the expected probabilities of these topics (9). Unlike traditional aggregation methods, this model allows a single record to encompass multiple topics simultaneously, making it particularly suitable for extracting research topics from regulations and policies (10). It can effectively automate the mining of research topics in a large number of policies, which is helpful for a better understanding of the research situation in a specific field. It can also provide a more objective evaluation of the topics and hot topics of policies, and is particularly suitable for topic extraction and evolution analysis of policy texts at a specific stage. So far, no scholar has conducted an analysis of policy evolution using the LDA topic model for TCMRAPs. In this study, we employ the LDA topic model as a key approach for topic identification. First, we determine the number of stages in the TCMRAPs process by analyzing the number of publications related to policies and regulations. Next, we examine the number of topics extracted by the LDA model at each stage. Finally, we explore the evolutionary trends of TCMRAPs by analyzing the keywords and prominent topics across the various stages.

This study aims to provide policymakers with valuable references for developing policies to promote the TCM industry. We utilize the LDA topic model to extract topics related to TCMRAPs. Through a detailed analysis of the content and significance of each topic, we aim to understand the research landscape of TCMRAPs from 1985 to 2024. The organization of this paper is as follows: Section 2 reviews the existing literature on TCMRAPs and the LDA topic model, emphasizing the limitations found in previous studies. Section 3 outlines the research design, including sample selection and methodology. Section 4 presents the analysis results of TCMRAPs, accompanied by a comparative analysis. Section 5 discusses the evolution of popular topics and policy topics. Finally, Section 6 summarizes the findings and presents conclusions and implications.

## 2 Literature review

In recent years, many scholars have actively explored the TCM policies. Some studies suggest a noticeable lack of alignment between policy tools and policy objectives, which has hindered the effectiveness of implementation. Consequently, there is a pressing need for a synergy between these policies and their associated tools (11). Researchers have employed advanced models, such as the TCM Development Policy, for quantitative assessments, including the PMC Index Model (12). Their analyses indicate a need for optimization in policy design, recommending a greater emphasis on the standardization and scientific foundation of policy formulation. Additionally, some scholars have also utilized text mining technology alongside the PMC Index Model to create an evaluation system that conducts quantitative comparisons of TCM innovation policies (13). Their findings suggest that the application of demand-side tools in these policies still requires enhancement. In more detailed studies, some scholars have systematically reviewed the registered varieties of Chinese patent medicines, examining the registration application and approval process. They have clarified the research direction for Chinese patent medicine enterprises (14). Through extensive literature review and analysis, it was discovered that, despite numerous studies addressing the registration and approval policies of TCM from various perspectives, many existing studies employ relatively outdated research methods (15). These methods are inadequate for accurately evaluating the evolution of these policies. Additionally, some studies face issues with sample selection, which prevents them from correctly screening policy sample information and, as a result, obtaining accurate conclusions. After thorough examination, it is suggested that TCM Regulatory Action Plans (TCMRAPs) should be categorized into four levels. The first level consists of national laws and regulations, such as the *Pharmaceutical Administration Law of the People’s Republic of China* and the *Pharmaceutical Registration Management Measures of the People’s Republic of China*. All individual and enterprise activities must comply with these laws. The second level includes classifications of drugs, with a specific focus on TCM. The relevant document for this level is the *Registration Classification and Application Materials Requirements* of TCM in Different Periods. The third level encompasses the *Technical Requirements Guidelines* related to the review and approval of TCM registration, which is where most studies tend to stop. Finally, the fourth level consists of the *Review and Approval Notices* pertaining to TCM registration.

In view of this, this study will systematically collect the policy documents related to the registration and approval of TCM from 1985 to 2024, use the LDA topic model to construct a comprehensive evaluation index system, in-depth explore the connotation of policy texts through quantitative analysis, accurately clarify policy objectives, and reveal the dynamic evolution path of policy topics. This study aims to provide a scientific basis for the optimization and improvement of the registration, review and approval policy of TCM, and help promote the sustained and high-quality development of the TCM industry.

## 3 Research design

### 3.1 Data sources, samples selection and study procedures

**Figure 1:**
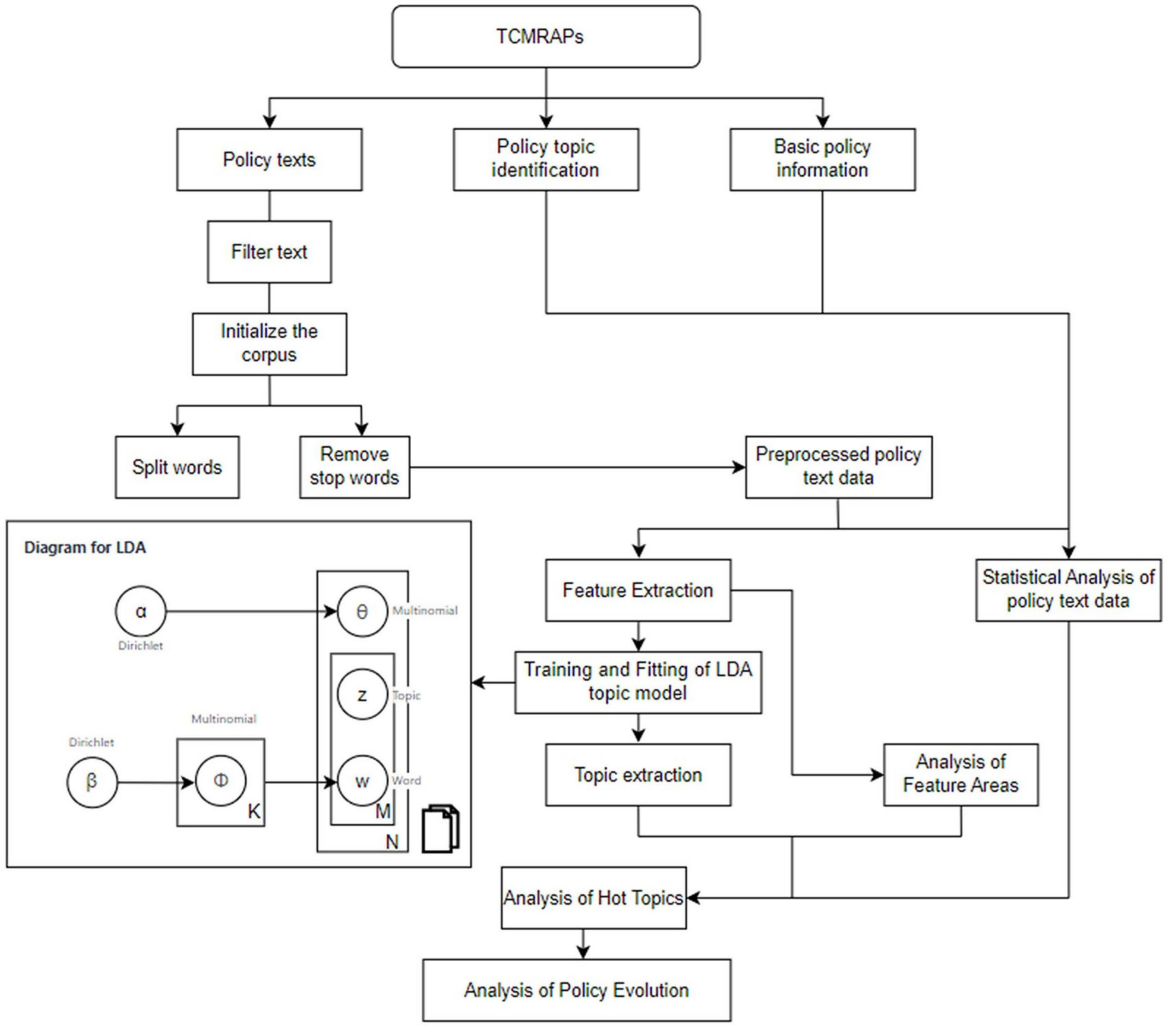
Study procedures.

In this study, the relevant policies on the review and evaluation of all TCMRAPs issued by the Chinese government/NMPA are reviewed. The time period selected for this article is from 1 July 1985, to 31 December 2024. Policy texts were mainly retrieved and obtained from the official portal website of China’s central government,^1^ the Law and Regulation Database of Peking University,^2^ the China Food and Drug Administration,^3^ China National Knowledge Infrastructure,^4^ and other common retrieval platforms. In addition, relevant Supplementary material were searched from Baidu, Google, Bing, and other websites. The selected policy is related to the registration, review, and approval policy of TCM. In addition to “TCM registration,” “TCM review,” “TCM approval,” “New medicine review,” “Drug registration,” the search scope was also expanded by entering keywords such as “TCM innovation” to ensure a comprehensive and careful search. The types of policies retrieved included laws, ordinances, methods, rules and requirements, opinions, announcements, guiding principles, and notifications. Then, the policies that were that were not related to TCMRAPs were excluded through manual screening. Due to the wide timeline span involved, these websites do not provide a detailed classification of these policies. The texts retrieved through keyword search include some that are not policies at all, but rather interviews by TV stations, interpretations of policies by celebrities, highlights of local governments, and so on. These policy documents are not issued at the national level and cannot be deleted through the procedures. Therefore, they need to be manually selected and deleted. Through this comprehensive collection, we ensured the comprehensiveness, authority, and reliability of the analysis results. The study procedures of this study are shown in Figure 1. Some key policies are shown in Table 1.

**Table 1:** TCMRAPs list.

No.	Policy title	Issuing agency	Date issued	Type
1	Notice on matters concerning the implementation of the measures for the examination and approval of new drugs	MH	1985	Notifications
2	Drug administration law of the People’s Republic of China	SC	1985	Laws
3	Measures for the examination and approval of new drugs	MH	1985	Methods
7	Supplementary provisions and explanations on TCM-related issues in the measures for the examination and approval of new drugs	MH	1987	Rules and Requirements
17	Regulations on the protection of traditional Chinese medicine (TCM) varieties	SC	1993	Ordinances
18	Guiding principles for clinical research of new traditional Chinese medicine (TCM) (trial implementation)	MH	1993	Guiding principles
49	Technical guiding principles for pilot-scale research of Traditional Chinese medicine (TCM) and natural medicines	CDE	2005	Guiding principles
50	Technical guiding principles for the research of preparations of traditional Chinese medicine (TCM) and natural medicines	CDE	2005	Guiding principles
51	Technical guiding principles for the pretreatment of raw materials of traditional Chinese medicine (TCM) and natural medicines	CDE	2005	Guiding principles
167	Suggestions on strengthening supply-side structural reform to promote the innovative development of traditional Chinese medicine (TCM)	SCNPC	2016	Opinions
190	Issuance of the “opinions on deepening the reform of the examination and approval system to encourage innovation in drugs and medical devices”	MCLSD	2017	Opinions
195	Traditional Chinese medicine (TCM) law of the People’s Republic of China	NATCM	2017	Laws
216	Announcement on adjusting the examination and approval procedures for drug clinical trials	NMPA	2018	Announcements
235	Announcement on issuing the “registration classification and declaration materials requirements for traditional Chinese medicine (TCM)”	NMPA	2020	Announcements
248	Measures for the administration of drug registration	SAMR	2020	Methods
253	Announcement on matters concerning the implementation of the 2020 edition of the “pharmacopoeia of the People’s Republic of China”	NMPA	2020	Announcements
359	Drug standard management measures	NMPA	2023	Methods
360	Announcement on the issuance of special provisions for the management of traditional Chinese medicine standards	NMPA	2024	Announcements
383	Key work tasks for deepening the reform of the medical and health system in 2024	SC	2024	Guiding principles

SC: State Council; SCNPC: Standing Committee of the National People’s Congress; SAMR: State Administration for Market Regulation; NMPA: National Medical Products Administration; NATCM: National Administration of Traditional Chinese Medicine; MH: Ministry of Health; MCLSD: Military Commission’s Logistics Support Department’s Health Bureau; CDE: National Medical Products Administration Center for Drug Evaluation.

### 3.2 Construction of LDA topic model

LDA topic model is a probabilistic generative model for unsupervised learning (16). The model architecture consists of three levels: document, topic, and word (17). The core idea is that a document is composed of multiple topics, each of which is composed of a specific set of words. In a document, each word occurs according to a specific probability distribution, so the topic of the document can be precisely characterized by word combinations with high probability (18). The joint distribution formula for the model is shown as Equation 1.


(1) $$p(\theta ,z,w|\alpha ,\beta )=p(\theta |\alpha )\prod_{n=1}^{N}p(z_{n}|\theta )p(w_{n}|z_{n},\beta )$$

where *θ* is the topic distribution, *α* and *β* are *a priori* parameters estimated based on actual experience, default values are generally used, and topic z and topic word w can be obtained by the Gibbs sampling algorithm (19). In this study, potential topics of TCMRAPs in the initial corpus were explored by importing LdaModel from genism model. Two results were obtained by analyzing the initial corpus through LDA topic modeling. The first result, which shows the optimal number of topics per stage, was used to explore how many topics TCMRAPs had in each stage. The second result shows the number of hot topics in each stage, which is used to explore the hot topics of TCMRAPs in each stage.

### 3.3 Determination of LDA topic model and number of topics

The first step is data preprocessing. In order to transform unstructured text data into a structured form that can be effectively processed and recognized by computers, this study uses the jieba module in Python to carry out word segmentation (Python 3.11 version, jieba module 0.42.1 version). By adding a custom dictionary, policy-related words can be accurately identified (20); At the same time, redundant words and pause words that may interfere with the accuracy of thematic analysis were eliminated with the help of a stop word dictionary (21). After that, the segmentation results are converted into vector patterns that can be computed and recognized by the LDA topic model. After literature research (22), the length of the term was set to be greater than 1, and the frequency was set to be greater than or equal to 3, so as to extract high-frequency keywords in the policy text of the TCMRAPs. The Second step is LDA topic model construction for TCMRAPs (23). The LdaModel module in gensim module (version 4.3.3) was used to build the topic model. This module can transform TCMRAPs words into word vectors, so as to clearly reveal the topic distribution of documents. When setting the parameters of LDA, the experience of previous research literature (18) was referred to, and factors such as frequency and topic contribution were comprehensively considered. The parameters *α* and *β* were set as 0.1 and 0.01 respectively, and the number of iterations was set as iters = 1,000 (10). In order to enhance the reproducibility of the experiment, we have added a random seed number, which is 42. There is a close correlation between the effect of LDA topic extraction and the number of potential topics K. In terms of determining the optimal number of topics K, there are generally the following three common methods (24): (1) Evaluation methods based on confusion: Confusion is one of the key indicators to evaluate the performance of language models. In the topic model, the smaller the confusion value of the model, the stronger the prediction ability of the model for the text, and the better the corresponding topic number K. However, when the number of topics K is selected solely based on the degree of confusion, the result of a large number of topics is often obtained, which leads to a high degree of overlap of the constituent words of topics. In addition, the correlation between confusion and human subjective judgment is low, and in some cases even shows a negative correlation. It follows that confusion is not the best measure of the number of topics. (2) Bayesian model-based method: This method mainly uses Gibbs sampling algorithm to determine the optimal number of topics. When it is difficult to sample directly, Gibbs sampling algorithm is able to approximately extract sample sequences from multivariable probability distributions. Although the calculation results of this method are relatively accurate, it has certain limitations in practical application due to the high complexity of its algorithm and the difficulty in judging the convergence of the LDA topic model. (3) Non-parametric processing method (25): the typical representative of this method is Hierarchical Dirichlet Processes (HDP). HDP can automatically train the appropriate parameters in a non-parametric form, without manually specifying the number of topics in advance, so as to effectively avoid the bias that may be introduced by manually setting the number of topics. However, this algorithm has the problem of high time complexity and relatively high computational cost. Considering the respective limitations of the above three methods, this study decided to use the combination of Perplexity and Coherence scores to select the optimal number of topics. The formula for the coherence score (16) is described as Equations 2, 3.


(2) $$\text{coherence}(V)=\sum_{(v_{i},v_{j})\in V}\text{score}(v_{i},v_{j},\varepsilon )$$

(3) $$\text{score}(v_{i},v_{j},\varepsilon )=\log\frac{p(v_{i},v_{j})+\varepsilon }{p(v_{j})}$$

In the formula, V denotes a set of words that characterize the topic. $\nu_{i}\;$ serving as the smoothing factor, is empirically set to 1. $\nu_{i}\;$ and $\nu_{j}\;$ represent any two distinct words within V, respectively. $p(\nu_{i},\nu_{j})$ stands for the co—occurrence probability of $\nu_{i}\;$ and $\nu_{j}\;$ . The coherence score has a positive correlation with sentence similarity, which is derived from calculating the co—occurrence recurrence of words in the sentence. Thus, a higher coherence score is indicative of a more favorable result. The calculation formula of perplexity is as Equation 4.


(4) $$\text{Perplexity}=\exp\{-\frac{\sum_{m=1}^{M}\log(w_{m})}{\sum_{m=1}^{M}N_{m}}\}$$

In the formula, *N* represents the document length, M represents the document set (20). At the same time, pyLDAvis module (version 3.4.1) was used to visualize the topic and verify whether the selection result of the topic was optimal.

**Figure 2:**
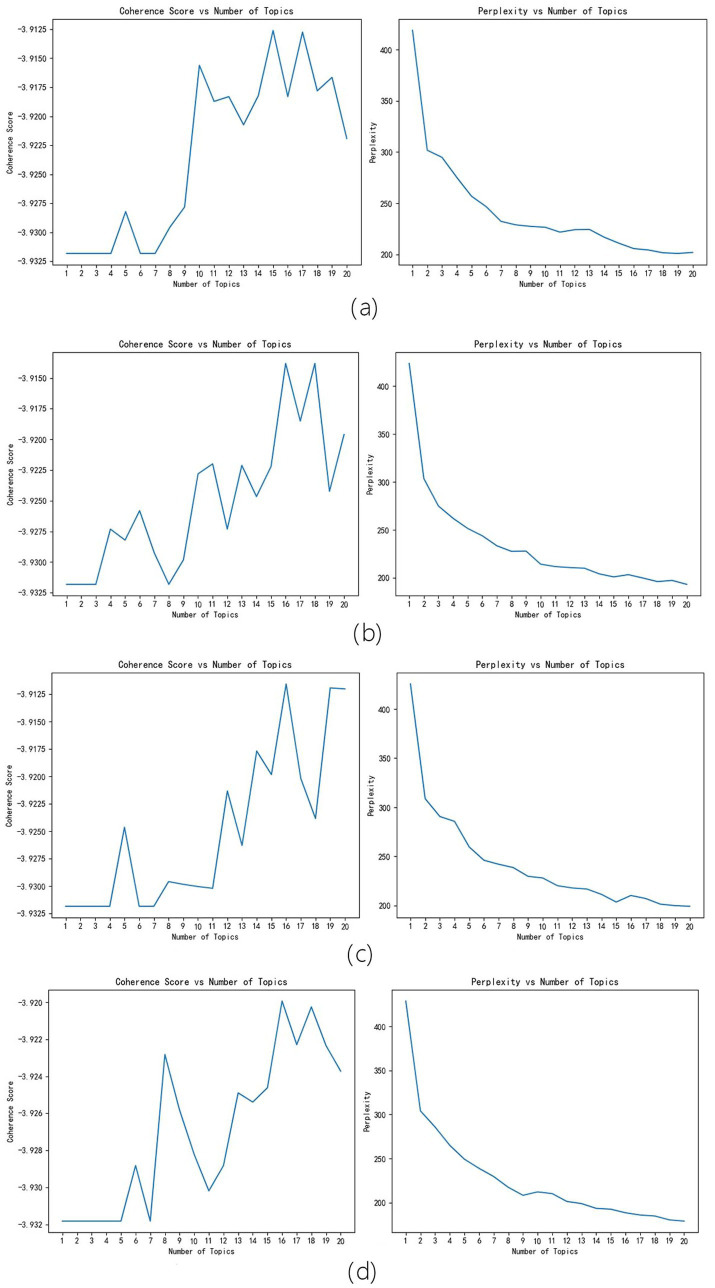
Random Seeds Robustness Tests Results. **(a)** correspond to random seed number 11 results, **(b)** correspond to random seed number 23 results, **(c)** correspond to random seed number 47 results, **(d)** correspond to random seed number 80 results.

In order to verify the robustness of the LDA model system, a random function was used to randomly generate four numbers as the random seed numbers. The four random seed numbers are 11, 23, 47, and 80. 11 correspond to Figure 2a, 23 correspond to Figure 2b, 47 correspond to Figure 2c, and 80 correspond to Figure 2d. It can be seen that different random seed numbers do not affect the results of the model – all results were 16 or 17. Therefore, the accuracy of the model is high.

### 3.4 Hot topic identification

The central concept in exploring research frontiers is “topic strength.” Topic strength refers to the extent to which specialists concentrate on particular research outputs (19). A higher topic strength signifies the topic is given more attention (17). Topic strength serves as a quantitative indicator that helps determine whether a research topic can be considered a state-of-the-art subject. In this study, when identifying frontiers using the LDA topic model, we propose that topic strength reflects the degree of importance that the issuing authorities (such as the government and the NMPA) attach to the topic. The higher the strength of a certain topic at a certain stage, the more it should be given attention at that stage. Specifically, topic strength can be calculated as the ratio of the sum of weights assigned to explored topics across all scientific reports to the total number of documents in the corpus. This relationship is represented in Equation 5.


(5) $$\theta_{j}=\sum_{d}\theta_{j}^{\left(d\right)}/M$$

In the formula, M represents the total number of documents (if contextually specific to a report—based corpus). $\theta_{j}\;$ denotes the overall topic intensity of topic j. $\theta_{j}\;$ refers to the weight assigned to topic j within document d, which is used to reflect the significance of the topic within that document. The larger the value of $\theta_{j}\;$ for a topic, the more prominent the proportion of that topic within the document set, and the greater the attention it garners. Thus, a topic with a relatively high $\theta_{j}\;$ value attracts significant focus in the document set. The average topic intensity value across all topics in the document set can be calculated using the formula. In this study, the topic intensity of each topic is calculated and compared with the average topic intensity of the current period, and the topic with greater than the average intensity is the hot topic.

### 3.5 Analysis of topic evolution

Topic evolution analysis is an important method to study the evolution of a topic in different time stages or between research stages. It can reveal the development context, trend, and key turning points of a subject field or a specific research topic. In this study, the core goal of topic evolution analysis is to identify the topic similarity in different stages, deeply analyze the correlation between the research topics of the TCMRAPs in different historical periods or policy backgrounds, and then construct the evolution path and mode of the topic. The topic similarity of this study was calculated using the cosine similarity method.

## 4 Results and analysis

### 4.1 Results of policy screening

**Figure 3:**
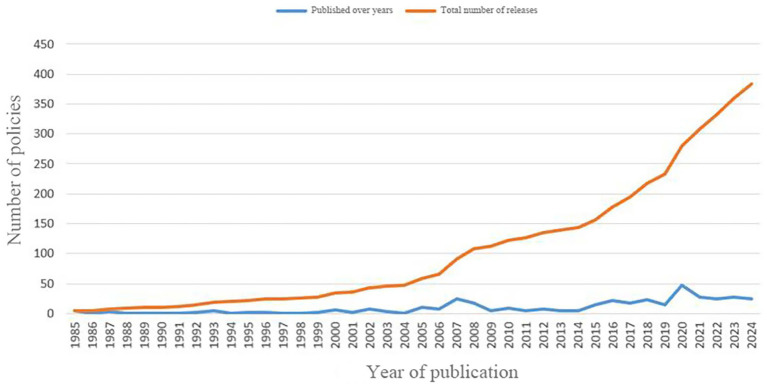
Policies numbers published in 1985–2024.

Through preliminary screening, a total of 603 texts were collected, and after policy screening, 383 texts were included in the final study. Among them, the notification policy (37. 34%) accounted for the largest proportion, followed by the guiding principle policy (22. 19%) as shown in Table 2. The amount and cumulative amount of policies line chart for each year are shown in Figure 3.

**Table 2:** Collect policies.

Policy type	Number	Proportion (%)
Laws	8	2.09
Ordinances	12	3.13
Methods	22	5.74
Rules and requirements	35	9.14
Opinions	37	9.66
Announcements	41	10.70
Guiding principles	85	22.19
Notifications	143	37.34

### 4.2 Results of time distribution of policy release

Based on the year of policy release and the actual development of TCMRAPs, this study divides the overall timeline into four key periods: the period of new drug approval measures, the embryonic period of drug registration management measures, the exploration period of drug registration management measures, and the deepening period of drug registration management measures. The specific division is based on the following: from 1985 to 2002, the programmatic document for drug approval in China was the *Measures for New Drug Approval.* Since its implementation in 1985, it has been revised and updated in 1992 and 1999, respectively. As can be observed from Figure 3, each revision or update leads to a temporary lift in the number of policy releases. However, the overall number of policy publications during this period was relatively low, so we grouped the years 1985 to 2001 into phase 1, the period of new drug approval measures. In 2002, the *Measures for Drug Registration Administration* was put into practice on a trial basis, marking a major change in the policy framework for the TCMRAPs in China. Since then, the method has been updated three times: it was formally implemented in 2005, updated in 2007, and updated again in 2020. As can be seen from Figure 3, there were significant peaks in the number of policy releases in 2007 and 2020. Based on this, in the period of the *Drug Registration Management Measures,* this study further divided the timeline into three stages: 2002 to 2006 was the embryonic period of the drug registration management measures, 2007 to 2019 was the stable implementation period of the drug registration management measures, and 2020 to 2024 was the deepening reform period of the drug registration management measures.

### 4.3 Results of topic extraction

#### 4.3.1 Determination of the number of policy topics

**Figure 4:**
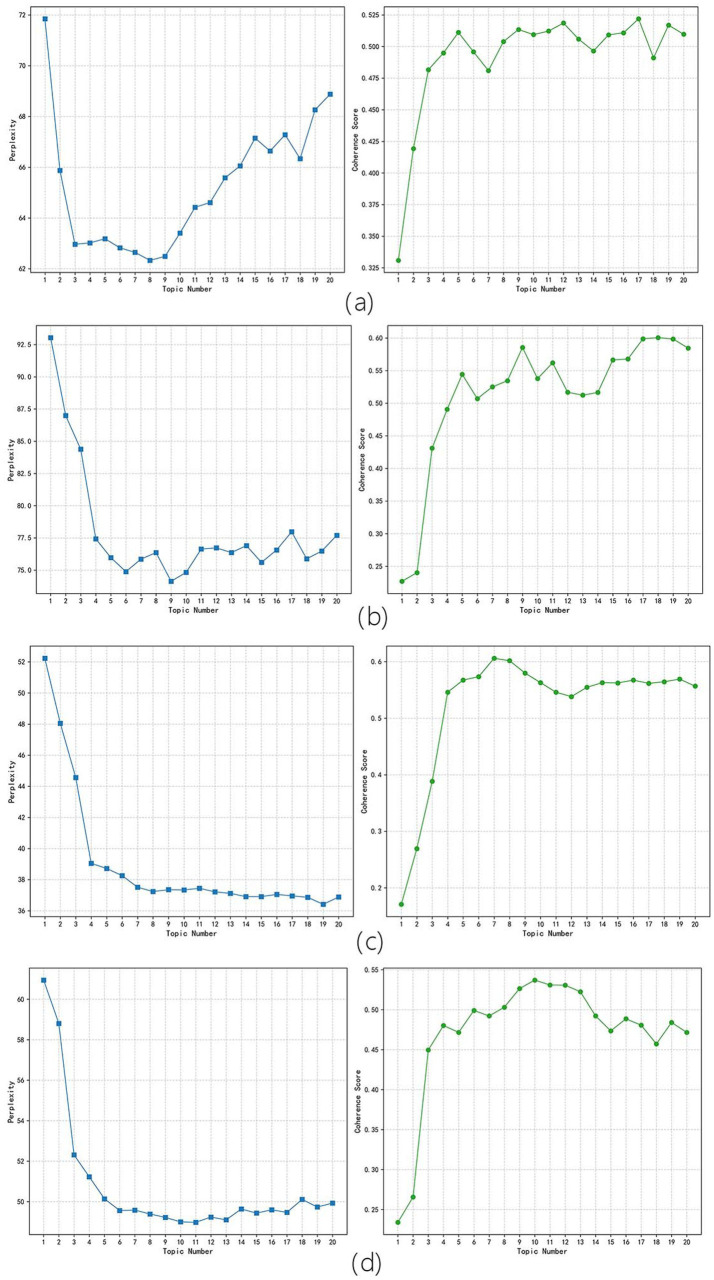
Perplexity and Coherence scores Results. **(a)** The period of new drug approval measures. **(b)** The embryonic period of drug registration management measures. **(c)** The stable implementation period of drug registration management measures. **(d)** The period of deepening reform of drug registration management measures.

**Figure 5:**
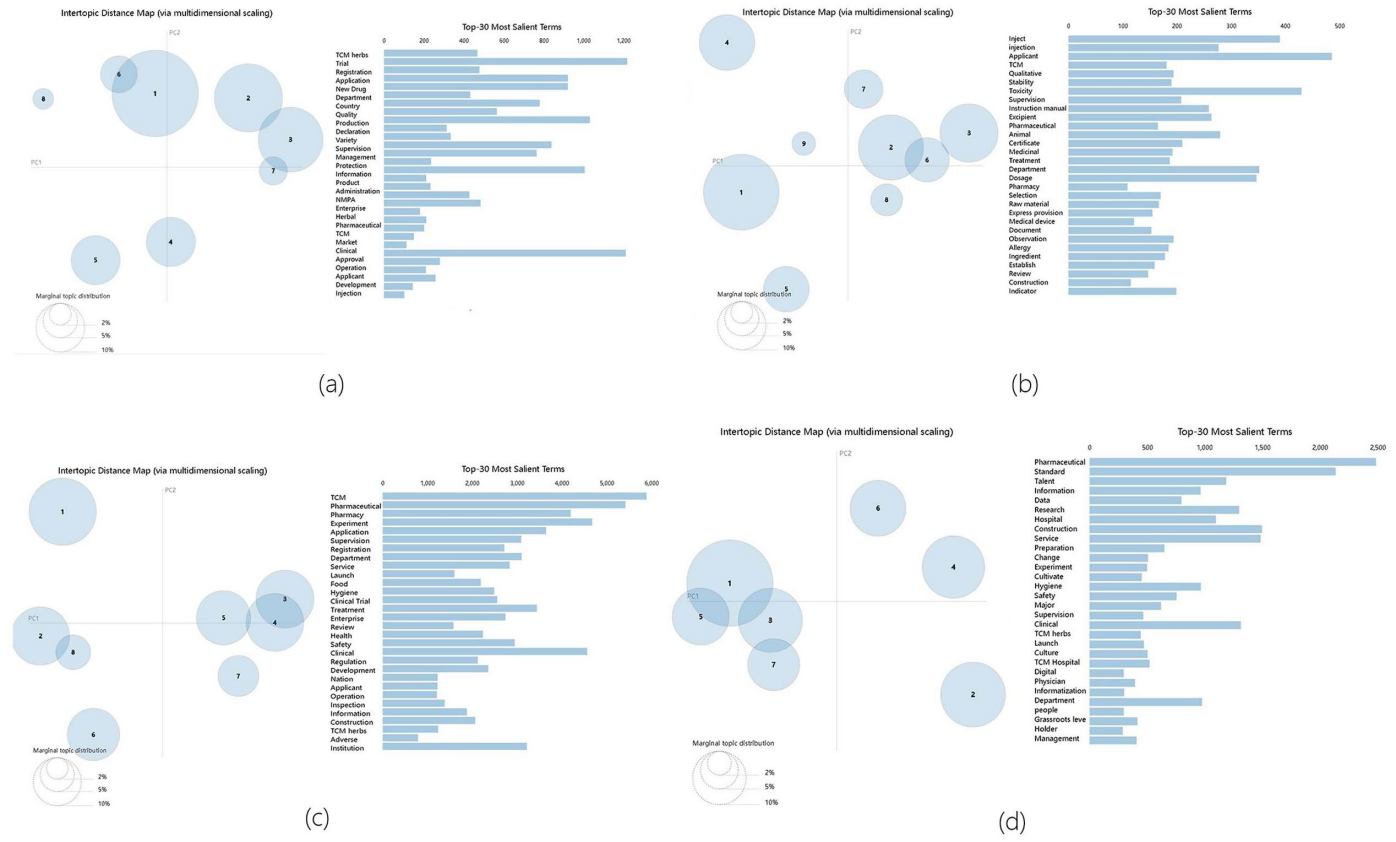
pyLDAvis visualization Results. **(a)** The period of new drug approval measures. **(b)** The embryonic period of drug registration management measures. **(c)** The stable implementation period of drug registration management measures. **(d)** The period of deepening reform of drug registration management measures.

In this study, the optimal number of topics was determined by analyzing the visual map, which combined the confusion curve and the pyLDAvis clustering result of the topic coherence curve. The lower the confusion value, the better the effect. If the difference in confusion was not significant, the topic coherence curve of the two was used to judge, and the greater the topic coherence value, the better the effect. As shown in Figure 4a corresponding to the period of new drug approval measures’ perplexity and coherence scores line chart, Figure 4b corresponding to the embryonic period of drug registration management measures’ perplexity and coherence scores line chart, Figure 4c corresponding to the stable implementation period of drug registration management measures’ perplexity and coherence scores line chart, and Figure 4d corresponding to the deepening reform period of drug registration management measures’ perplexity and coherence scores line chart. The optimal number of topics for the four periods was determined as follows: 8, 9, 8, and 7. Using pyLDAvis for visualization mapping, as shown in Figure 5a corresponding to the period of new drug approval measures’ pyLDAvis visualization picture, Figure 5b corresponding to the embryonic period of drug registration management measures’ pyLDAvis visualization picture, Figure 5c corresponding to the stable implementation period of drug registration management measures’ pyLDAvis visualization picture, and Figure 5d corresponding to the deepening reform period of drug registration management measures’ pyLDAvis visualization picture. It can be found that topics in each stage are interrelated but not overlapping, indicating that the number of topics is selected ideally.

#### 4.3.2 Hot topic identification

**Figure 6:**
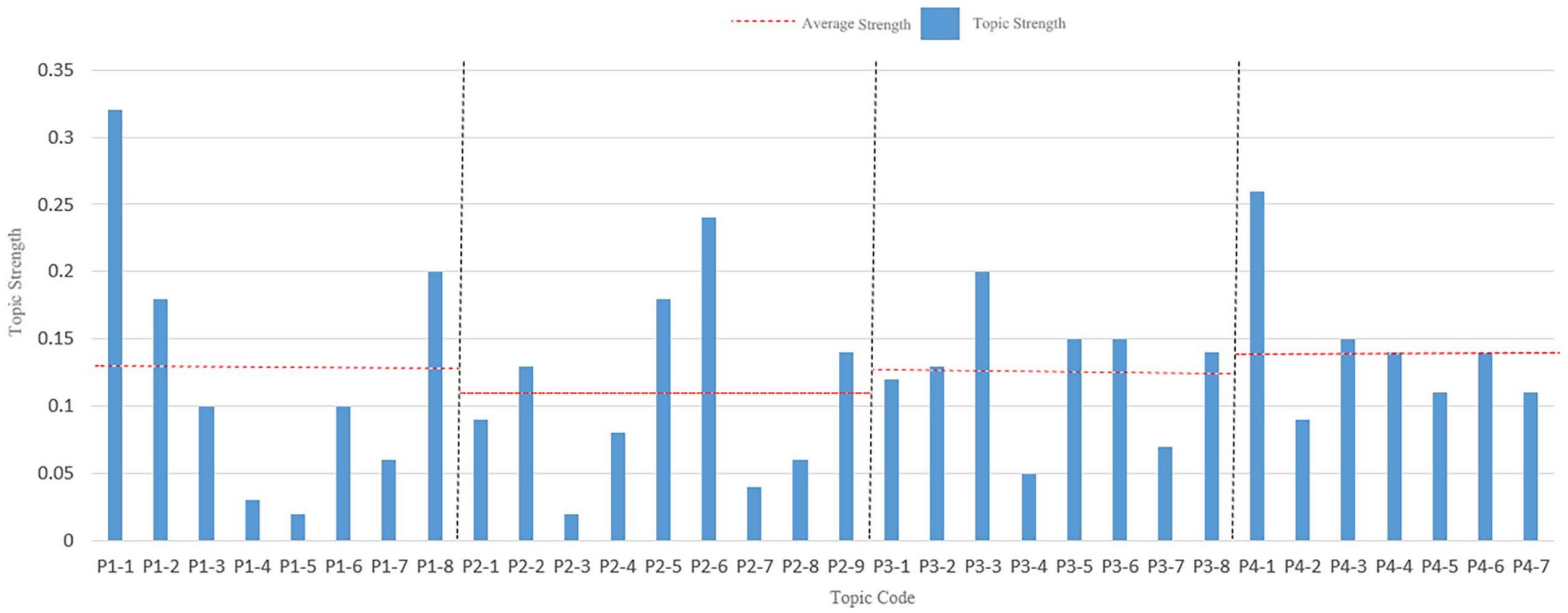
Hot topics of TCMRAPs for each period.

Focusing on the policy hotspots in each period, to clarify the development of the TCMRPAPs. Hot topics were identified by comparing the intensity value of each topic with the average intensity value. First, the topics were coded, the four periods were named P1-P4, and the topics of each period were distinguished by adding a number after each period, such as the first topic of the first period, coded as P1-1, and so on. The results are shown in Figure 6. The height of the bars is the thematic intensity value, and the horizontal dashed line is the average thematic intensity value for each period. There are significant differences in popular topics across each period. The topics with higher than average topic strength were set as hot topics, and the number of hot topics selected in the final four periods was 3, 4, 5, and 4, respectively.

The topic names of hot words in all topics in all periods were assigned, and the results of the assignment are shown in Table 3. Due to space reasons, only five hot keywords that can best reflect the meaning of each topic are listed.

**Table 3:** Topic names of hot words.

Period	Topic code	Hot topic or not	Topic identify	Hot keywords (top 5)
Period of new drug approval measures	P1-1	Yes	Construction of regulatory system for TCMRAPs	Application, supervision and management, declaration, research, clinical
	P1-2	Yes	Clinical research of TCM	Clinical trial, subject, efficacy, adverse reaction, control
	P1-3	No	Development of TCM industry	Product, development, technology, enterprise, market
	P1-4	No	Technical standards of TCM	Injection, method, determination, quality standard, production
	P1-5	No	Patent protection of TCM	Patent, protection, variety protection period, committee
	P1-6	No	Market supervision of TCM	Enterprise, administration, operation, institution, supervision and management
	P1-7	No	Approval of new TCM	New drug, production, approval, ministry of health, enterprise
	P1-8	Yes	R & D of new TCM	Data, trial, new drug, research, preparation
Embryonic period of drug registration management measures	P2-1	No	Registration submission documents of TCM	Certification document, institution, attachment, document, approval
	P2-2	Yes	Market supervision of TCM	Supervision, medical, market, establishment, product
	P2-3	No	Management of raw materials and excipients of TCM	Instruction Manual, national medical products administration, raw material, excipient, release
	P2-4	No	Refinement of injection technology for Chinese	Injection, component, excipient, indication, qualitative
	P2-5	Yes	Registration submission documents of TCM	Writing, note, description, efficacy, brief
	P2-6	Yes	Registration application of TCM	Applicant, department, approval, opinion, sample
	P2-7	No	Quality control of TCM	Quality, stability, selection, investigation, indicator
	P2-8	No	Standardization of TCM	Standard setting, statutory, purification, standard, tradition
	P2-9	Yes	Non-clinical research of TCM	Toxicity, animal, dosage, allergy, irritation
Stable implementation period of drug registration management measures	P3-1	No	Improvement of TCMRAPs	Department, supervision and management, enterprise, regulation, institution
	P3-2	Yes	Upgrade of TCM Industry	Safety, construction, supervision, system, product
	P3-3	Yes	Scientific evaluation of TCM	Change, process, preparation, clinical trial, safety
	P3-4	No	Registration documents of TCM	Marketing authorization, inspection, adverse, report, holder
	P3-5	Yes	Optimization of the TCM evaluation procedures	Application, registration, clinical trial, applicant, data
	P3-6	Yes	Inheritance and cultural promotion of TCM	Inheritance, construction, characteristic, talent, innovation
	P3-7	No	Production management of TCM	Work, medical, government, institution, procurement
	P3-8	Yes	Management of TCM services	Service, medical, health, wellness, ethnic
Deepening reform period of drug registration management measures	P4-1	Yes	Characteristic development route of TCM	Construction, service, culture, promotion, innovation
	P4-2	No	Digital transformation of TCM	Information, data, safety, service, TCM hospital
	P4-3	Yes	Research guidance of TCM	Classic formula, change, quality, safety, development
	P4-4	Yes	Development of the TCM Industry	Health, service, technology, specialty, medical institution
	P4-5	No	Construction of the talent system of TCM	Talent, training, construction, education
	P4-6	Yes	Improvement of regulations and strengthening of legal responsibilities for traditional Chinese medicine	Supervision and management, production, holder, institution, regulation

The first stage was the period of new drug approval measures (1985–2001); this stage marked the beginning of the classification registration of new TCM. At the same time, the relevant laws for supervision and management of traditional Chinese medicine were also being gradually established. For instance, in the *Drug Administration Law of the People’s Republic of China* issued in 1985, it states: “To strengthen the supervision and management of drugs, ensure drug quality, enhance drug efficacy, guarantee the safety of drug use by the people, and safeguard the health of the people, this law is hereby formulated.” This indicates that Construction of Regulatory System for TCMRAPs is being established. The *Guideline for Clinical Research of new TCM* issued in 1993 stated that “the purpose of clinical research on new traditional Chinese medicines is to evaluate the therapeutic or preventive effects of a certain drug on certain diseases and its safety. The research conclusion should answer the questions of whether the drug has clinical practical value for the aforementioned diseases and how to use it, in order to determine whether the drug can be widely used in clinical practice.” This indicates that as the development progresses, traditional Chinese medicine clinical research has gradually gained attention from regulatory authorities. Through policy regulations, enterprises are guided to conduct standardized research. The establishment of the regulatory process belongs to the construction of the regulatory system, while new drug registration falls within the scope of the research and development of new traditional Chinese medicines. During the research process, the keyword “clinical” is particularly prominent. Therefore, combining the hot topic key words, the three hot topics identified in the first stage are: Construction of Regulatory System for TCMRAPs, Clinical Research of TCM, R & D of New TCM.

The second stage was the embryonic period of drug registration management measures (2002–2006). Although the *Drug Registration Management Measures* were just promulgated at this stage, the regulatory system had been established for 16 years. However, with the listing of a large number of drugs, some registration application issues arose, such as the significant change in the classification of traditional Chinese medicine registration in 2002, which was expanded to 11 categories, supplementing the categories missing in the previous version, and the descriptions of each category became clearer. This indicates that the attitude of national regulatory authorities towards traditional Chinese medicine registration applications remained important during this stage, and the system was gradually improved in the regulatory process, including the development of guidance principles for non-clinical research and the standardization of application materials. At the same time, for the already listed drugs, further market supervision also requires policy support for management, such as the *Notice on Issuing the National Special Action Plan for Rectifying and Standardizing the Drug Market Order* issued by the State Council in 2006. This also indicates that the national regulatory authorities attached importance to market supervision at this stage. Therefore, by combining the keywords of the hot topics, the four hot topics of this stage can be obtained: Market Supervision of TCM, Registration Submission Documents of TCM, Registration Application of TCM, and Non-Clinical Research of TCM.

The third stage is the stable implementation period of drug registration management measures (2007–2019). By 2007, the *Drug Registration Management Measures* had been in effect for 6 years, and during this period, they underwent two updates. The classification of traditional Chinese medicine registration was revised from 11 categories to 9 categories. At the same time, Chinese drugs began to align with international standards during this period. China’s traditional Chinese medicine industry began to upgrade, such as the *Health Development Outline for the ‘11th Five-Year Plan’* in 2008, which clearly stated “continuously improve the independent innovation ability of traditional Chinese medicine, promote the modernization of traditional Chinese medicine and the sustainable development of the industry.” The *Health Development Outline for the ‘11th Five-Year Plan’* also emphasized “the ‘11th Five-Year Plan’ is a crucial stage for building a moderately prosperous society in all respects and building a socialist harmonious society, and it is also an important period for implementing the scientific development concept. Health reform and development face good opportunities and also shoulder heavy tasks.” This indicates that the evaluation of traditional Chinese medicine in China is following a scientific path, and the process is also following an optimized path. During the same period, the *Several Opinions on Supporting and Promoting the Development of Traditional Chinese Medicine* and the *Notice on Printing and Distributing the Functional Allocation, Internal Institutions and Staffing Scheme of the State Medical Administration* also indicate that China’s supporting services and management for traditional Chinese medicine are following the process of optimization. Therefore, based on the keywords of the hot topics, we can identify the five main topics of this stage: Upgrade of TCM Industry, Scientific Evaluation of TCM, Optimization of the TCM Evaluation Procedures, Inheritance and Cultural Promotion of TCM, and Management of TCM Services.

The final stage is the period of deepening reform of drug registration management measures (2020–2024). During this stage, both the system and the registration management measures have been in operation for an extended period. During this period, both enterprises and regulatory authorities have accumulated sufficient experience. In the 2020 version of the *Drug Registration Management Measures*, the classification of TCM was simplified into four categories, and policies related to expedited approval were introduced into the process. The *Notice on several measures for accelerating the Development of TCM Characteristics*, released in 2021, indicates that China has begun to follow its own unique development path while balancing regulations and responsibilities. Various guiding principles, including Technical Guidelines for each stage of new drug research in TCM, were gradually promulgated in 2020, indicating that China’s guiding documents are continually improving and can better guide enterprises in developing new drugs. At the same time, new technologies have emerged, including AI. In 2020, the *Opinions of the National Medical Products Administration on Promoting the Inheritance and Innovation Development of Traditional Chinese Medicine* was issued by the state. In 2024, the *Notice of the State Council on Comprehensively Deepening the Reform of Drug and Medical Device Supervision* emphasized the need for the traditional Chinese medicine industry to continue developing, transitioning from its previous conventional model to “digitalization” and “digitization.” Therefore, based on the keywords of this stage, the four hot topics of this stage are obtained: Characteristic Development Route of TCM, Research Guidance of TCM, Development of the TCM Industry and Improvement of Regulations and Strengthening of Legal Responsibilities for Traditional Chinese Medicine.

By examining the number of topics and hot topics across the four stages, China’s policies have a broad scope and encompass a wide range of aspects, basically encompassing all fields of the TCM industry.

#### 4.3.3 Topic evolution analysis

**Figure 7:**
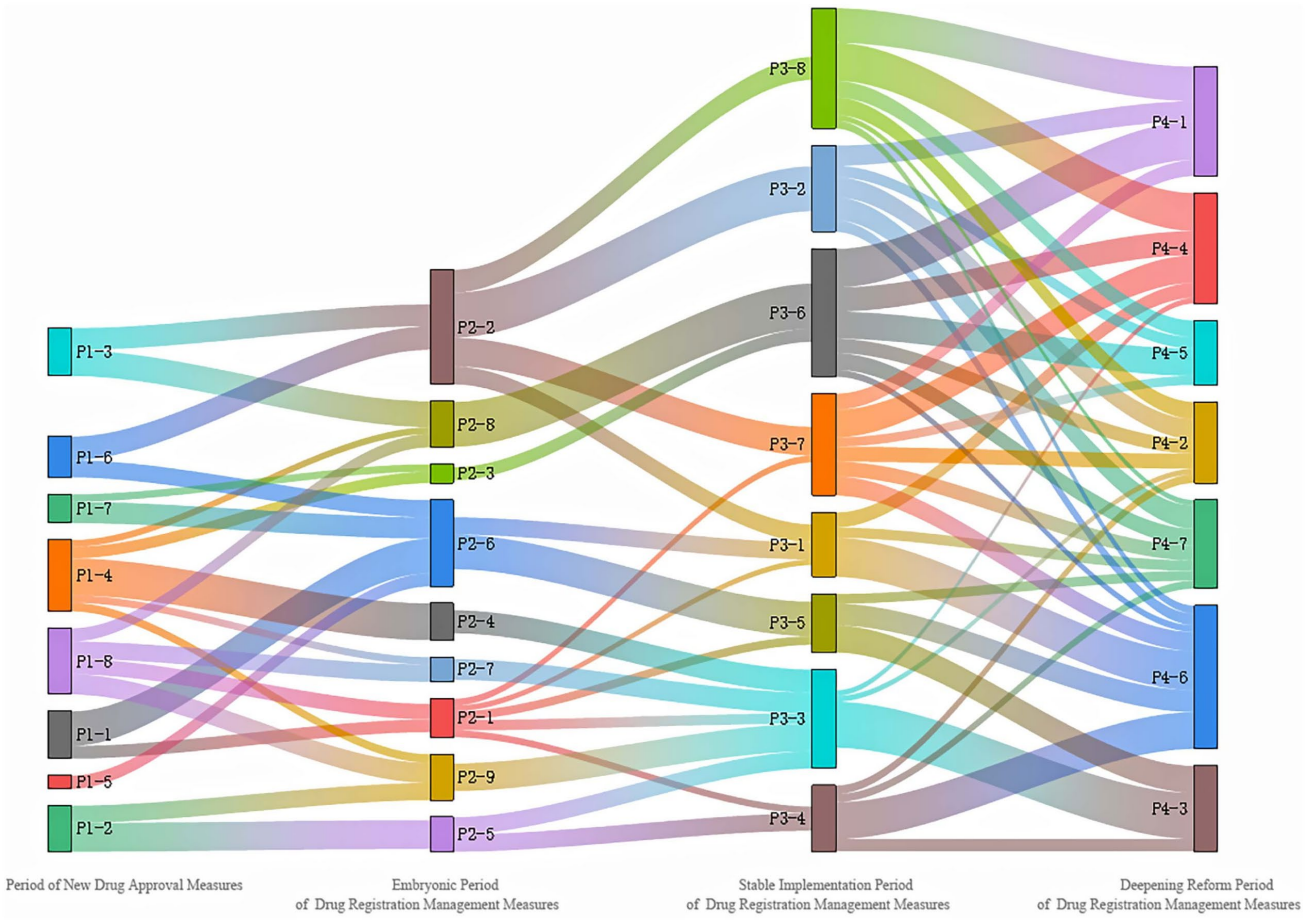
Sankey diagram of topic relevance for each period.

At the same time, in order to further explore the topic evolution process of the four stages, the topic similarity between different stages is calculated according to the toy-word probability distribution obtained by the LDA topic model. According to relevant research (14), the two topics with similarity greater than 0.3 are regarded as the existence of evolutionary relationship, and the topic evolution path of the registration and approval policy of TCM. Sankey diagram of topic relevance is as shown in Figure 7. Among them, each element block corresponds to each topic, and the line between topics represents the flow direction and connection between topics. The thickness of the line represents the similarity degree. The thicker the line, the closer the evolutionary relationship between topics. It is clear that based on Figure 7, the evolution path of China’s TCMRAPs is extremely complex and intricate. Each topic does not exist independently but rather functions as a hub in a network, interacting closely and providing support with other topics.

## 5 Discussion

### 5.1 Discussion of hot topics in each period

#### 5.1.1 Discussion of hot topics in the period of new drug approval measures

During the period of new drug approval measures, three hot topics have attracted much attention: “Construction of Regulatory System for TCMRAPs,” “R & D of New TCM,” and “Clinical Research of TCM.” At the forefront is the topic of “Construction of Regulatory System for TCMRAPs.” In 1985, the *Drug Administration Law of the People’s Republic of China* was enacted, marking a significant milestone as China’s drug supervision and administration officially entered the legal framework. Following this, the 1989 *Measures for the Implementation of the Drug Administration Law* established a strong legal foundation for developing the regulatory system for TCM. In 1999, with the release of a series of policy documents such as *Matters Related to the Implementation of the Approval Measures for New Drugs*, *New Drug Protection* and *Technology Transfer Provisions* by the State Medical Products Administration, the regulatory system of TCM in China was further improved, and a systematic and scientific regulatory framework was gradually constructed. The topic of “R & D of New TCM” was in a sub-core position. The introduction of the *Measures for New Drug Approval* in 1985 marked a crucial milestone in the establishment of this policy focus. For the first time, new drugs were categorized into three types: TCM, Western medicine, and biological products. This classification created a basic framework for the approval of new TCMs. As a result, the registration of new drugs in China is now officially conducted through three distinct pathways, as outlined by the regulations. For the TCM part, in that same year, the Ministry of Health issued a notice regarding the implementation of new drug approval measures. This notice established the approval process for TCM for the first time through administrative instructions. In 1992, the Ministry of Health issued the *Provisions on the Revision and Supplementary Provisions concerning TCM*. This updated document clarified the ambiguities present in the first version of the Measures for the *Approval of New Drugs* and further enhanced the approval system for new Chinese medicines. In 1999, the former State Medical Products Administration (SDA) updated the *Approval Measures for New Drugs*. This update further optimized the approval process and regulatory mechanisms, enhancing the science and standardization of the approval for new TCM. This series of policy changes highlights the country’s focus on TCM research. It also shows that during the initial phase of establishing the TCM R&D Policy Framework, policymakers recognized the importance of TCM research. The topic of “clinical research of TCM” has also attracted much attention in this period. The *Guiding Principles for Clinical Research of New TCM*, published in 1993, established a solid scientific framework for the clinical research of TCM. These principles clarified the basic requirements and operational specifications necessary for conducting clinical research in this field. They also provided a scientific foundation for the clinical evaluation of new TCM treatments. During this period, the introduction of these policy documents showcased a dynamic balance between fostering the development of TCM and ensuring risk control within the regulatory framework at its initial stages. This multi-faceted approach not only encouraged innovation and growth within the TCM industry but also prioritized the safety and efficacy of medications. As a result, it established a strong foundation for the sustainable development of the TCM sector and effectively advanced its modernization.

#### 5.1.2 Discussion of hot topics in the embryonic period of drug registration management measures

During the embryonic period of drug registration management, four hot topics have attracted much attention: “application for registration of TCM,” “registration submission documents of TCM,” “non-clinical research of TCM,” and “market supervision of TCM.” The topics of “application for registration of TCM” and “registration submission documents of TCM” occupy the core position. In 2002, the State Council (SC) issued the Regulations of the *People’s Republic of China on TCM* and the *Administrative Measures for Drug Registration*. This marked a significant milestone, signifying the transition of China’s drug approval system from the *New Drug Approval Measures* to the *Drug Registration Administrative Measures* era. TCM, being an important category of drugs, is included in the registration management scope defined by these *Drug Registration Management Measures*. The management of drug registration has been gradually refined over time. In 2002, the State Food and Drug Administration issued the *Registration Application and Application Information Requirements for TCM and Natural Medicine*. This document standardized the registration process for TCM and outlined specific information requirements, providing clear guidelines for the registration management of TCM. In 2003, the State Food and Drug Administration clarified the vague description in the *Measures for Drug Registration and Administration* by issuing the *Notice on the Issuance of Supplementary Provisions on Drug Registration and Administration*. In 2005, the *Administrative Measures for Drug Registration* were revised, along with the *Requirements for Registration and Application of TCM and Natural Medicine*. These updates indicated that this period marked the beginning stages of implementing the *Drug Registration Management Measures*. The entire system was transitioning from *New Drug Approval Measures* to a framework more aligned with drug registration management. The topic of “non-clinical research of TCM” occupied a sub-core position during this period, reflecting that the country began to place greater emphasis on the quality control of TCM at this stage. In 2005, the Center for Drug Evaluation of the National Medical Products Administration issued a series of guiding principles, including the *Technical Guidelines for Pilot Studies of TCM and Natural Drugs*. These documents provide detailed specifications for the pre-registration non-clinical research of TCM and thus play a key role in the quality control of TCM in China. The topic of “market regulation of TCM” also attracted much attention, and its hot keywords included “supervision, medical, market, establishment, product,” etc., indicating that China had attached great importance to the market regulation of TCM registration in this period. In 2006, the SC issued two important policy documents: the *Notice of the General Office of the SC on the Issuance of the Special Action Plan for the Rectification and Regulation of the Drug Market* and the *Notice on Strengthening the Supervision and Inspection of the Professional Market of TCM Materials*. These documents provided robust support for the regulation of the TCM market. These four key topics are highly significant during this stage, which marks a crucial transition from implementing new drug approval measures to adopting drug registration management measures. This phase also represents the initial implementation of drug registration management. In the broader context, TCMRAPs have been in operation for some time. The shift in prominent topics reflects the moment when two important guiding documents are revised, indicating a change in policymakers’ attitudes toward policy priorities, and highlighting potential issues that may arise during the operation of China’s TCMRAPs.

#### 5.1.3 Discussion of hot topics in the stable implementation period of drug registration management measures

During the stable period of drug registration management, five hot topics have attracted much attention: “scientific evaluation of TCM,” “optimization of the TCM evaluation process,” “inheritance and cultural promotion of TCM,” “management of TCM services,” and “upgrade of the TCM industry.” Among them, “scientific evaluation of TCM” and “optimization of the TCM evaluation process” both focused on optimizing TCMRAPs and could be discussed together. They occupied a significant position in this period. In 2007, the SC issued the *National 11th Five-Year Plan for Food and Drug Safety*, which outlined development goals for that planning period. The plan aimed to gradually improve the food and drug regulatory system and mechanism, establishing a comprehensive legal and regulatory framework over five years. It also emphasized the importance of enhancing the quality of the supervision team to strengthen the ability to enforce laws effectively. Additionally, the plan called for the reinforcement of infrastructure, improvement of technology and equipment, and a significant increase in food and drug safety standards and testing technologies. We will substantially improve the order of food and drug production and Distribution, effectively curb illegal and criminal activities of producing and selling fake and substandard food and drugs, and reduce food and drug safety accidents. In the same year, the new *Measures for Drug Registration Administration* were formally implemented, and the registration administration of the TCM stepped into the track of standardization, thus enhancing the safety, effectiveness, and quality of TCM products. With the implementation of the new method, a series of supporting guiding principles have been updated successively, such as the *Regulations on the Administration of Special Approval for New Drug Registration*, the *Format and Requirements for Clinical Research Review of TCM and natural drugs*, the *format and requirements for pharmaceutical Research Review of TCM and natural drugs*, and the *Technical Guiding Principles for general Pharmacological Research of TCM and natural drugs*. The current measures indicate that the TCM industry in China is experiencing robust growth. Various policies are actively supporting the industry’s development, and the registration and evaluation policy framework for TCM is continually improving. Overall, the registration and evaluation system for TCM in China is becoming increasingly stable. “inheritance and cultural promotion of TCM,” “management of TCM services,” and “upgrade of the TCM industry” were in the secondary position. They all belong to TCM industry optimization topics and can be studied together. The optimization of the TCM industry encompasses various aspects, including the heritage and innovative development pathways of TCM, cultural promotion, management of TCM medical services, and talent training. These keywords highlight our country’s efforts in promoting the implementation of the policy, while also paying attention to the upgrading of industrial supporting facilities. The *Notice of the Bureau of TCM on the printing and Distribution of the Guidelines for the Cultural Construction of the TCM Hospitals* was issued by the Bureau of TCM, which helped the optimization of the TCM industry from the aspects of talent and infrastructure. Although these policies may not seem directly related to the registration of TCM, they actually support the effective implementation of the registration, review, and approval system for TCM. In 2019, the Central Committee of the Communist Party of China and the SC issued the *Opinions of the Central Committee of the Communist Party of China and the State Council on Promoting the Development of the Inheritance and Innovation of TCM*. This document once again emphasized the importance of advancing the inheritance and innovation of TCM in China, highlighting the renewed focus on the evaluation and approval system for TCM. During this period, China’s drug registration management measures were further enhanced. With strong support from national policies, the TCM industry made significant progress. The registration, examination, and approval system for TCM became more stable, laying a solid foundation for the inheritance, innovation, and development of TCM in China.

#### 5.1.4 Discussion of hot topics in the deepening reform period of drug registration management measures

During the deepening reform period of drug registration management measures, four hot topics emerged: the “characteristic development route of TCM,” “research guidance of TCM,” “development of the TCM industry,” and “improvement of regulations and strengthening of legal responsibilities of TCM.” The characteristic development route of TCM plays a significant role. In 2020, to better implement the *Opinions of the Central Committee of the Communist Party of China* and the *State Council on Promoting the Inheritance and Innovative Development of TCM*, the National Food and Drug Administration issued the *Implementation Opinions of the State Food and Drug Administration on Promoting the Inheritance and Innovative Development of TCM*. This marked a significant turning point in the research and development of new TCM in China. “research guidance of TCM” occupied a secondary position. In 2020, China updated *Administrative Measures for Drug Registration* once more. A series of requirements and guiding principles were revised in the *Administrative Measures for Drug Registration*. These updates indicate that the country has established more rigorous standards for the safety and quality of TCM products. Several important documents were released, including the *Technical Guiding Principles for Research on the Production Process of TCM Compound Preparations*, the *Technical Guiding Principles for Research on Samples for Toxicological Studies of New TCM*, and the *Technical Guiding Principles for Research on the Quality Control of Medicinal Materials for New TCM*. In 2023, the State Food and Drug Administration issued the *Notice of the State Food and Drug Administration on Several Measures to further Strengthen the scientific supervision of TCM and Promote the Inheritance, Innovation and Development of TCM*, which further deepened the development route of Chinese medicine characteristics. “development of the TCM industry” and “improvement of regulations and strengthening of legal responsibilities of TCM” have also attracted much attention. This indicates that China made significant efforts to develop the TCM industry during this period, paying special attention to cultivating talent in the process of industrial development. These policies encompass the personnel planning for the TCM registration system and an overview of the TCM examination and approval system construction. It shows that our country has been optimizing the supporting personnel and processes related to the registration, examination, and approval policy in ancient times, during the drug administration period, to adapt to the development route of TCM in our country.

### 5.2 Discussion of policy topic evolution results

#### 5.2.1 Discussion of the evolution of TCM regulatory system

Starting in 1985, China’s pharmaceutical supervision entered its initial and improvement stages. The official promulgation of the *Pharmaceutical Administration Law of the People’s Republic of China* marked the beginning of legal oversight in this field. In 1989, the I*mplementation Measures of the Pharmaceutical Administration Law* were issued, followed by a series of related documents in 1999. These developments further enhanced the supervision system for TCM. They gradually established a systematic and scientific framework for oversight, providing a solid foundation for the standardized growth of the TCM industry. However, there are still significant limitations in the supervision. For instance, the quality standards for traditional Chinese medicine processing techniques are vaguely defined, and there is overlap in the regulatory responsibilities among different departments, which has led to uneven quality of Chinese herbal decoctions in some regions. As a result, the expected goal of “systematic supervision” has not been fully achieved (26). By 2002, the review and approval system for TCM was undergoing significant transformation and improvement. The SC issued the *Regulations of the People’s Republic of China on TCM* and the *Measures for Drug Registration*. This transition marked a significant shift in China’s drug approval system, from the *New Drug Approval Measures* to the *Measures for Drug Registration*. During this period, the application process and data requirements for TCM registration became more detailed and standardized, thereby enhancing the scientific rigor and precision of the approval process for TCM. After 2007, a phase of optimization and systematization began. The new *Regulations for Drug Registration* were implemented, accompanied by a series of supporting guidelines. Since then, the policy framework for the registration and evaluation of TCM in China has been continuously improved. However, there is still a drawback of “emphasizing procedures over substance,” such as a relatively low requirement for the mechanism research of traditional Chinese medicine compound preparations, which is inconsistent with international drug regulatory standards (27). There is also the problem of overly lengthy classification of traditional Chinese medicines. In 2020 and 2023, the National Food and Drug Administration issued relevant opinions and notifications that further advanced the reform of the evaluation and approval system for TCM. In the 2020 version of the *Drug Registration Management Measures*, the registration classification for traditional Chinese medicine has been simplified to four categories. These measures effectively promoted the inheritance and innovation of TCM, leading to the continued Development of a more optimized and systematic review and approval process. The evolution of the TCM regulatory system has shifted from a decentralized approach to a more systematic one. Such as the “fingerprint pattern” quality control method for traditional Chinese medicine registration has been recognized by some foreign regulatory agencies (28). However, some methods and theoretical systems have not yet gained international recognition, such as the syndrome differentiation and treatment system (29). Therefore, it is necessary to establish a more effective connection mechanism between “traditional experience” and “international standards.”

#### 5.2.2 Discussion of the development of TCM industry

From 1985 to 2006, as new drugs were being approved and drug registration management was still developing, the introduction of policy documents not only advanced the development of TCM but also ensured the efficacy of these drugs. This laid a solid foundation for the sustainable development of the TCM industry. However, in the field of traditional Chinese medicine injections, due to the rapid approval process, adverse events were overlooked (30). Some enterprises also neglected the accumulation of clinical evidence, thereby laying hidden risks for subsequent quality issues. After 2007, the TCM industry entered a phase of significant expansion, marked by the stable and enhanced implementation of drug registration management measures. The government not only focused on promoting policies but also continuously optimized the supporting infrastructure for the TCM industry, including talent development and facility construction. Additionally, there was an emphasis on the inheritance and cultural promotion of TCM, the management of TCM medical services, and the modernization of the TCM industry to ensure its comprehensive development. At the same time, this stage also emphasizes the examination of drug safety. During this stage, the industrial growth is still mainly driven by scale expansion. Nevertheless, there are still many issues in this stage: for instance, the integration of intangible cultural heritage of traditional Chinese medicine processing techniques with modern production technologies is insufficient; some traditional processes lack standardized transformation; and the traditional Chinese medicine formulas mainly come from natural sources (plants, animals, minerals), resulting in quality differences at each stage (15). These problems make it difficult to meet the requirements of large-scale production, resulting in the dual challenges of a “transmission gap” and “quality fluctuations.” Following 2020, a new phase of quality improvement began for TCM. The National Food and Drug Administration issued guidelines to promote the Research and development of new TCM products, which reached an unprecedented level. These guidelines emphasized a unique development path for TCM, directing research efforts towards innovation and supporting the growth of the TCM industry alongside the establishment of a talent development system. As a result of this policy support, the TCM industry could advance significantly, and the quality of TCM products improved markedly. The trajectory of the TCM industry’s development has shifted from simply expanding scale to improving quality. However, to achieve high-quality development, we still need to address further core issues such as “modernization of traditional techniques” and “insufficient international competitiveness of products.” In particular, we need to make breakthroughs in “accumulation of evidence-based data”—through international multi-center clinical trials, to provide globally recognized evidence to support the effectiveness and safety of traditional Chinese medicine, and promote the industry to upgrade from “domestic dominance” to “international participation”.

#### 5.2.3 Discussion of TCM innovation

From 1985 to 2001, the focus was on the technical verification stage. During the implementation of new drug approval measures, the *Guidelines for Clinical Research of New TCM* issued in 1993, along with other regulatory documents, established a robust technical framework for TCM research. This framework promoted the standardization and scientific rigor of TCM clinical research, paving the way for the innovation and development of new TCM therapies. However, in this stage, there is still a problem of “insufficient scientificity” in innovation. For instance, the research on the mechanism of action of traditional Chinese medicine compound prescriptions mostly stays at the “*in vitro* experiment” level, lacking in-depth studies such as *in vivo* metabolism and target validation. It is difficult to explain the essence of the efficacy of traditional Chinese medicine from a scientific perspective, and there is a significant gap compared to the requirements of “evidence-based medicine” (25). Starting in 2002, the emphasis shifted to standardizing non-clinical research. The *Technical Guidelines for Clinical Trials of TCM and Natural Drugs*, issued in 2005, further defined the standards for non-clinical research prior to the registration of TCM products. This strengthened the quality control of TCM in China and provided stricter quality assurances for TCM innovation. However, in practice, there is still a situation of “disconnection between standards and practice”: for instance, the toxicological evaluation methods for traditional Chinese medicine compound prescriptions still follow the research paradigm of single-component drugs of Western medicine, failing to fully consider the characteristics of traditional Chinese medicine as “having multiple components and multiple targets,” which results in some traditional prescriptions without toxic side effects being unable to pass the non-clinical review due to “not meeting the evaluation standards of Western medicine,” thereby restricting the innovation and transformation of traditional experience (25). After 2007, the focus transitioned to a continuous innovation-driven stage. Relevant policy documents issued by the National Food and Drug Administration in 2020 and 2023 have further stimulated the innovation of new TCM research and the review and approval processes. These developments enable the TCM industry to continue evolving through innovation. The progress of TCM innovation has shifted from empirical practice based on experience to methods based on scientific evidence. However, with the continuous emergence of new technologies and the iterative improvement of existing ones, the innovation process of traditional Chinese medicine in our country needs to keep pace with current trends. It should shift from the conventional model to being more “digital” and “digitally intelligent.” Some research has already explored the application of new technologies in various aspects of the traditional Chinese medicine industry. Only in this way can the efficacy of traditional Chinese medicine provide “traditional characteristics & modern science” as dual evidence, and promote the innovation of traditional Chinese medicine to move from “domestic recognition” to “global evidence-based” recognition (31).

## 6 Conclusions and implications

### 6.1 Conclusion

In this study, the LDA topic model was used to analyze the policy evolution of TCMRAPs from 1985 to 2024. Not only the hot topics in the period of new drug approval measures (1985–2001), the embryonic period of drug registration management measures (2002–2006), the stable implementation period of drug registration management measures (2007–2019) and the deepening reform period of drug registration management measures (2020–2024), The evolution trend of TCM registration and approval policy in the past decade was analyzed from an overall perspective, and three conclusions were drawn.

First, from the breadth of topic distribution, the scope of concern of the TCMRAPs in China is extremely broad, covering all key areas of the development of the TCM. The construction and improvement of the examination and approval system of the TCM, the promotion of the scale expansion and quality upgrading of the TCM industry, and the guidance of the TCM innovation from experience-driven to scientific and evidence-based, all demonstrate the systematic support of policies for the TCM.

Second, from the comparative analysis of hot topics, it can be seen that the hotspots of Chinese medicine registration, review, and approval policies show their uniqueness in each stage. In the period of new drug examination and approval measures, the construction of the examination and approval system of the TCM, the examination and approval of new Chinese medicine, and the clinical research of the TCM were carried out. In the initial stage, the measures focused on the registration application of the TCM, the standard of application data, non-clinical research, and market supervision. In the stable period, it focuses on optimizing the scientific evaluation of the TCM, inheriting its culture, medical service management, and industrial upgrading. In the deepening period, the characteristics of TCM development, new drug research guidance, industry development, and talent system construction were highlighted. These phased changes are accurate responses and positive adjustments to changing medical needs, continuous technological development, and the inheritance and promotion of the TCM culture, which have effectively promoted the steady improvement of TCM service quality and the continuous improvement of the medical system.

Thirdly, based on the analysis of the evolution process of the topic, with the passage of time, the TCMRAPs have shown a development trend from the basic framework to standardization and refinement. The early policy was devoted to building the basic framework, such as the initial establishment of the regulatory system, the basic promotion of industrial development, etc. Then, it gradually turned to the standardization of the process and refinement requirements, such as the refinement of the registration application process and application data, the improvement of non-clinical research standards, etc. Recently, more emphasis has been placed on comprehensive development, including characteristic development routes, new drug research guidance, industry deepening, and talent system construction. This trend reflects that in the process of formulating the TCM policies, the state has always taken improving the overall quality of service and medical standards as the core goal and has made continuous efforts and progress.

### 6.2 Implications

Based on this study, we believe that the findings provide valuable insights for optimizing the structural design of TCMRAPs in China. Additionally, it establishes a reusable framework for policy optimization at both the theoretical and practical levels.

From a domestic perspective, TCMRAPs serve as the central government’s key support for the sustainable development of TCM. The policy evolution identified in this study can serve as a reference for policymakers in formulating subsequent policies. It can also provide future development ideas for TCM enterprises. For policymakers, it is essential to continue adhering to the trend of standardization and refinement in development. Given the broad scope of TCM-related policies, there is a need to refine the policy system further, filling regulatory gaps and strengthening coordination between different policy documents. For example, as TCM increasingly integrates with modern medical research and international standards, policies should be more targeted to guide TCM innovation in line with evidence-based medicine requirements while preserving its unique cultural characteristics. Moreover, policymakers should maintain a dynamic adjustment mechanism. Drawing on the experience of previous policy adjustments in response to changing medical needs and technological development, future policies should be more proactive in anticipating emerging challenges, such as the Impact of artificial intelligence on TCM research and the globalization of TCM trade. For the TCM enterprises, these policies imply opportunities for transformation and upgrading. Enterprises should closely follow the policy orientation and increase investment in R&D, focusing on cultivating professional teams proficient in both traditional TCM theory and modern pharmaceutical technology to meet the requirements of scientific evaluation and quality improvement.

From an international perspective, the policy evolution research method proposed in this study applies to other countries seeking to enhance the level of traditional medicine or cultural heritage. Currently, the classification system for herbal medicines in the European Union is similar to the Chinese classification system for traditional medicine, and such a classification can better manage drug registration (32). Therefore, the European Union is particularly well-suited to using the same analytical methods to study policy evolution. Many other countries worldwide face the shared challenge of balancing the protection of traditional knowledge with the advancement of modern innovation. Examples include the standardization of Ayurvedic medicine in India and the regulated application of African traditional herbs. They can draw on the methods of this study to identify any unreasonable aspects in the evolution of their own policy topics over time and make timely corrections. They can also draw on the topics and hot topics covered by the Chinese traditional medicine registration policy to fill in the topics of their own drug registration policies, enabling the rapid development of the drug registration system. At the same time, they can also draw on the classification system of Chinese registration policies. The significance of this study lies not only in improving TCM development in China but also in offering important references for other countries looking to promote the preservation and advancement of their own traditional medicine and cultural heritage.

## Data Availability

The original contributions presented in the study are included in the article/Supplementary material, further inquiries can be directed to the corresponding author.

## Footnotes

^1^ https://www.gov.cn/

^2^ https://www.pkulaw.com/

^3^ https://www.nmpa.gov.cn/

^4^ https://www.cnki.net/
